# Plasma Elaidic Acid Level as Biomarker of Industrial *Trans* Fatty Acids and Risk of Weight Change: Report from the EPIC Study

**DOI:** 10.1371/journal.pone.0118206

**Published:** 2015-02-12

**Authors:** Véronique Chajès, Carine Biessy, Pietro Ferrari, Isabelle Romieu, Heinz Freisling, Inge Huybrechts, Augustin Scalbert, Bas Bueno de Mesquita, Dora Romaguera, Marc J. Gunter, Paolo Vineis, Camilla Plambeck Hansen, Marianne Uhre Jakobsen, Françoise Clavel-Chapelon, Guy Fagherazzi, Marie-Christine Boutron-Ruault, Verana Katzke, Jasmine Neamat-Allah, Heiner Boeing, Ursula Bachlechner, Antonia Trichopoulou, Androniki Naska, Philippos Orfanos, Valeria Pala, Giovanna Masala, Amalia Mattiello, Guri Skeie, Elisabete Weiderpass, Antonio Agudo, Jose Maria Huerta, Eva Ardanaz, Maria Jose Sánchez, Miren Dorronsoro, Jose Ramon Quirós, Ingegerd Johansson, Anna Winkvist, Emily Sonested, Tim Key, Kay-Tee Khaw, Nicolas J. Wareham, Petra H.M. Peeters, Nadia Slimani

**Affiliations:** 1 Nutrition and Metabolism Section, International Agency for Research on Cancer, Lyon, France; 2 Department of Epidemiology and Biostatistics, The School of Public Health, Imperial College London, London, United Kingdom; 3 Department For Determinants of Chronic Diseases (DCD), National Institute for Public Health and the Environment (RIVM), Bilthoven, The Netherlands; 4 Department of Gastroenterology and Hepatology, University Medical Centre, Utrecht, The Netherlands; 5 Department of Social & Preventive Medicine, Faculty of Medicine, University of Malaya, Kuala Lumpur, Malaysia; 6 Department of Public Health, Section of Epidemiology, Aarhus University, Aarhus, Denmark; 7 INSERM, Centre for research in Epidemiology and Population Health (CESP), U1018, Nutrition, Hormones and Women’s Health team, Villejuif, France; 8 University Paris Sud, UMRS 1018, Villejuif, France; 9 Institute Gustave Roussy, Villejuif, France; 10 The German Cancer Research Center (DKFZ), Heildelberg, Germany; 11 Department of Epidemiology, German Institute of Human Nutrition, Potsdam-Rehbruecke, Nuthetal, Germany; 12 Hellenic Health Foundation, Athens, Greece; 13 Department of Hygiene, Epidemiology and Medical Statistics, University of Athens Medical School, Athens, Greece; 14 Epidemiology and Prevention Unit, IRCCS Foundation, National Cancer Institute, Milan, Italy; 15 Molecular and Nutritional Epidemiology Unit, Cancer Research and Prevention Institute-ISPO, Florence, Italy; 16 Department of Clinical and Experimental Medicine, Federico II University, Naples, Italy; 17 Department of Community Medicine, University of Tromsø-The Arctic University of Norway, Norway; 18 Instituto de Investigacion Sanitaria de Palma (IdISPa) and CIBER Fisiopatología de la Obesidad y Nutrición (CIBEROBN), Spain; 19 Unit of Nutrition, Environment, and Cancer, Catalan Institute of Oncology-ICO, IDIBELL, L’Hospitalet de Llobregat, Barcelona, Spain; 20 Department of Epidemiology, Murcia Regional Health Council, Murcia, Spain; 21 CIBER Epidemiology and Public Health CIBERESP, Spain; 22 Navarre Public Health Institute, Pamplona, Spain; 23 Andalusian School of Public Health, Granada, Spain; 24 Public health Direction and Biodonostia- CIBERESP, Basque Regional Health Department, San Sebastian, Spain; 25 Public Health Directorate, Asturias, Spain; 26 Department of odontology, Umeå University, Sweden; 27 Department of Internal Medicine and Clinical Nutrition, Sahlgrenska Academy, University of Gothenburg, Sweden; 28 Department of Clinical Sciences in Malmö, Nutrition Epidemiology, Lund University, Malmö, Sweden; 29 The Cancer Epidemiology Unit, University of Oxford, Oxford, United Kingdom; 30 Clinical Gerontology Unit, Department of Public Health and Primary Care, Institute of Public Health, University of Cambridge, United Kingdom; 31 MRC Epidemiology Unit, Institute of Metabolic Science, Addenbrooke’s Hospital, Cambridge, United Kingdom; 32 Julius Center for Health Sciences and Primary Care, University Medical Centre Utrecht, Utrecht, The Netherlands; University of Basque Country, SPAIN

## Abstract

**Background:**

Few epidemiological studies have examined the association between dietary *trans* fatty acids and weight gain, and the evidence remains inconsistent. The main objective of the study was to investigate the prospective association between biomarker of industrial trans fatty acids and change in weight within the large study European Prospective Investigation into Cancer and Nutrition (EPIC) cohort.

**Methods:**

Baseline plasma fatty acid concentrations were determined in a representative EPIC sample from the 23 participating EPIC centers. A total of 1,945 individuals were followed for a median of 4.9 years to monitor weight change. The association between elaidic acid level and percent change of weight was investigated using a multinomial logistic regression model, adjusted by length of follow-up, age, energy, alcohol, smoking status, physical activity, and region.

**Results:**

In women, doubling elaidic acid was associated with a decreased risk of weight loss (odds ratio (OR) = 0.69, 95% confidence interval (CI) = 0.55-0.88, p = 0.002) and a trend was observed with an increased risk of weight gain during the 5-year follow-up (OR = 1.23, 95% CI = 0.97-1.56, p = 0.082) (p-trend<.0001). In men, a trend was observed for doubling elaidic acid level and risk of weight loss (OR = 0.82, 95% CI = 0.66-1.01, p = 0.062) while no significant association was found with risk of weight gain during the 5-year follow-up (OR = 1.08, 95% CI = 0.88-1.33, p = 0.454). No association was found for saturated and *cis*-monounsaturated fatty acids.

**Conclusions:**

These data suggest that a high intake of industrial *trans* fatty acids may decrease the risk of weight loss, particularly in women. Prevention of obesity should consider limiting the consumption of highly processed foods, the main source of industrially-produced *trans* fatty acids.

## Introduction

The worldwide increase in obesity, especially among young-age populations and low socio-economical groups, has largely been driven by the global nutrition transition that promotes a chronic energy imbalance, typically characterized by increases in the consumption of highly processed foods, sugar-sweetened beverages, refined carbohydrates, salt, and saturated and *trans* fatty acids (TFA) [1–3], with genetic factors likely modifying individual susceptibility to these dietary factors [4]. This change is coupled with lifestyles leading to reduced daily energy expenditure (e.g. increased television watching, less physical activity, more sedentary behaviors). Energy imbalance leads to storage of excess energy in adipocytes, which exhibit both hypertrophy and hyperplasia, endoplasmic reticulum stress, and mitochondrial dysfunction, leading to increased intracellular release of adipokines, free fatty acids, and inflammatory mediators that cause adipocyte dysfunction [4].

Several epidemiological studies have examined the association between dietary fatty acids and weight gain, but the epidemiological evidence remains scarce and inconsistent [5]. Specifically, there is still limited evidence that increased intake of industrial TFA may result in weight gain [6]. One of the major limitations in epidemiological studies relates to the imprecision in estimating fatty acid intake using traditional dietary questionnaires. Moreover, the conversion of food items into their fatty acid content is exceptionally complex for numerous reasons, including the variation of fatty acid composition within the same food according to cooking methods and industry supply, and the incompleteness of food composition tables, particularly regarding TFA isomers. In contrast, biomarkers of dietary fatty acids offer objective, qualitative measures of bioavailable amounts of some specific fatty acids irrespective of the source and quality of foods [7, 8]. Specifically, we recently showed that plasma phospholipid elaidic acid concentrations, the main TFA isomer occurring during partial hydrogenation of vegetable oils and found in a myriad of industrial foods, were positively correlated to the intake of highly processed foods within the European Prospective Investigation into Cancer and Nutrition (EPIC) cohort [9]. In this regard, the use of biomarkers of dietary industrial TFA and highly processed foods in epidemiological studies could provide key insights into the relationship between industrial TFA and weight gain.

Thus, the main objective of this study was to investigate the association between baseline plasma phospholipid fatty acid concentrations, with a particular focus on TFA from industrial food sources, and subsequent weight change within the EPIC-PANACEA (Physical Activity, Nutrition, Alcohol, Cessation of Smoking, Eating out of Home and Obesity) study.

## Materials and Methods

### Population

The rationale and design of the EPIC study have been previously reported [10, 11]. The EPIC cohort consists of more than 500,000 subjects distributed among 23 centres in 10 European countries (Denmark, France, Greece, Germany, Italy, the Netherlands, Norway, Spain, Sweden and United Kingdom). Between 1992 and 2000, country-specific dietary questionnaires (food frequency questionnaires or diet history questionnaires), standardized lifestyle, anthropometric data and blood samples were collected at baseline from the study participants. Blood samples were collected from most participants at baseline (1992–1998). In each of the 23 centres, blood samples of at least 30mL were drawn from most participants and stored at 5–10°C protected from light and transported to local laboratories for processing and aliquoting. The only exceptions were the EPIC-Oxford centre (United Kingdom) where blood samples were collected from a network of general practitioners and transported to a central laboratory by post, and centres in Sweden and Denmark where blood was aliquoted within one hour of drawing. In all countries, except Denmark and Sweden, blood was separated into 0.5mL fractions (serum, plasma, red cells and buffy coat for DNA extraction). Each fraction was placed into straws, which were heat sealed and stored under liquid nitrogen. In Denmark, blood fraction aliquots of 1.0mL were stored locally in Nunc tubes at -150°C under nitrogen vapour. In Sweden, samples were stored in -80°C freezers.

The EPIC-PANACEA study is a subcohort of EPIC where repeated measures of anthropometry are available, which aimed at determining predictors of body weight and subsequent changes in weight [12].

### Ethics Statement

All participants gave written or oral informed consent. The study was approved by the International Agency for Research on Cancer Ethical Review Committee and by local ethical committees at the participating centres.

### Selection Criteria

Plasma phospholipid fatty acid were measured in a sub-analysis of the EPIC study [7], for which detailed and standardized 24-hour dietary recalls (24-HDRs) were collected [13, 14]. For the purpose of these analyses, 16 geographical regions were designated by grouping some of the 23 participating EPIC centers together: France (Paris and surroundings), Northern Italy (Varese/Turin), Central Italy (Florence), Southern Italy (Ragusa/Naples), Northern Spain (San Sebastian, Navarra, Asturias), South-Eastern Spain (Murcia), Southern Spain (Granada), Greece (Athens and other regions), Northern Sweden (Umeå), Southern Sweden (Malmö), Denmark (Aarhus and Copenhagen), UK (Oxford, the health conscious group, vegans and ovo-lacto-vegetarians), UK (Cambridge, the General population), The Netherlands (Utrecht and Bilthoven), former East Germany (Potsdam), and South-West Germany (Heidelberg).

Except for France, where only women were recruited, 100 men and 100 women were randomly selected from each of the 16 regions, with equal numbers of subjects selected for each season at which their blood sample was collected. Thirty percent of samples were fasting. In total, 3,003 subjects were finally randomly selected for participation in this study from those who completed information on FFQ and 24-HDRs, and those with available blood samples.

The exclusion criteria for the present study were length of follow-up equals to 0, extreme reported energy intake (<1% and >99% percentile of the ratio of reported energy intake estimated through country-specific dietary questionnaires to estimated energy requirement), missing information on weight or height at baseline or extreme anthropometric measurements (height<130cm, body mass index<16kg/m^2^), missing weight at follow-up, missing information on lifestyle (tobacco or alcohol consumption), pregnancy, or chronic disease (prevalent diabetes, cancer or cardiovascular disease) at enrollment. The final population consisted of 1,998 individuals (54% women). Finally, elaidic acid measured through gas chromatography was not well separated in 53 chromatograms, leading to further exclusions and a total number of 1,945 individuals retained in this analysis.

### Assessment of Anthropometric Measures and Weight Change

Body weight and height were measured at baseline according to standardized procedures previously described [15]. Weight was measured to the nearest 0.1kg and height was measured to the nearest 0.1, 0.5, or 1.0cm depending on the center, without shoes. The body mass index (BMI) was calculated as body weight in kilograms divided by squared height in meters (kg/m^2^).

At follow-up (ranging from 2 to 11 years), information on weight was collected through questionnaires in all centers, except in the Bilthoven (The Netherlands) and Cambridge (UK) centers. In the Bilthoven center, weight was measured by trained staff for 57 persons, and self-reported values were collected from 56 persons. In the Cambridge center, weight was measured by trained staff. No follow-up data were available from Ragusa and Turin centers in Italy.

As the follow-up times differed by center, we calculated the weight change at 5 years (kg/5y) as (weight at follow-up—weight at baseline) x 5/years of follow-up. The weight at 5 years, determined adding the weight change at 5 years to the weight at baseline, and the BMI at 5 years were also obtained. The outcome measure used in our analysis was the percent of weight change at 5 years, computed as (weight at 5 years minus weight at baseline/weight at baseline) x100, and expressed as a percentage.

### Laboratory Analysis

Details on plasma phospholipid fatty acid measurements through gas chromatography were previously provided [16]. Samples of the same sex and age category were ordered randomly within analysis batches, and each batch included one subject from each participating centre and one sample from a standard pool for the quality control. Briefly, lipids were extracted from 200μl plasma, phospholipids were purified by solid-phase extraction, fatty acid methyl esters were formed by transmethylation of the phospholipids and analyzed by gas chromatography on a 30-m polar column. The relative amount for each fatty acid was expressed as the percentage of total area. The laboratory analysis method allowed for the analysis of twenty-two individual fatty acids with a chain length between 14 and 22 carbons belonging to different fatty acid classes: saturated fatty acids, monounsaturated fatty acids including one *trans* isomer, elaidic acid (*trans* 18:1n-9), n-3 polyunsaturated fatty acids and n-6 polyunsaturated fatty acids. Analytical quality control was carried out by the daily use of a standard quality control plasma (*n* = 137). The coefficients of variation (CV) for fatty acids were previously provided [7]. They ranged from 1.91% for major peaks such as stearic acid to 12.75% for minor peaks such as α-linolenic acid. CV was 5.38% specifically for elaidic acid. All analyses were performed at the IARC-WHO laboratory.

### Statistical Analysis and Data Treatment

The association between plasma phospholipid fatty acids and percent change of weight at 5 years was investigated using a multinomial logistic regression model. The percent change of weight at 5 years was categorized according to tertiles, i.e. for individuals showing a 5-year percent variation <-1.6%; between-1.6% and 2.8%; > 2.8%. Odds ratios expressing the risk of increasing (decreasing) weight at 5 years were computed comparing the top (bottom) tertile category versus the second, which was used as the reference category. The exposure variables were fatty acid concentrations, and were modeled as continuous variables, after log 2 transformation to express the variation in risk of percent of weight gain and weight loss associated to doubling the concentration level. Models were adjusted by length of follow-up (years), age (years), energy intake (kcal/day), alcohol intake (g/day), smoking status (never, former, current), physical activity (inactive, moderately inactive, moderately active, active), and region. Analyses were carried out for women and men separately.

Statistical tests were 2-sided, and p-values <0.05 were considered significant. All statistical analyses were performed using SAS statistical software (version 9.4, SAS Institute Inc, Cary, NC, USA).

## Results

The median follow-up time was 5.0 years (range 2.5–9.9 years) among women and 4.9 years (range 2.7–9.9 years) among men. Table 1 presents the baseline characteristics of the study population across sex-specific tertiles of plasma phospholipid elaidic acid level defined on the whole sample (i.e. including all studied regions), expressed as percentage of total fatty acids.

**Table 1:** Baseline characteristics of the study population across sex-specific tertiles of plasma phospholipid elaidic acid level.

	**Elaidic acid (%)**							
	***Men (869)***				***Women (1077)***			
	**< 0.12**	**[0.12–0.20]**	**≥ 20**	***p-value*** ^***a***^	**< 0.12**	**[0.12–0.20]**	**≥ 20**	***p-value*** ^***a***^
	431	254	184		217	395	465	
								
**Mean age** (yrs)	54.4 (0.3)	54.9 (0.4)	53.5 (0.4)	*0.16*	55.7 (0.4)	54.6 (0.3)	53.6 (0.3)	*<.0001*
**Mean weight** (kg)	81.5 (0.5)	80.3 (0.7)	77.9 (0.8)	*0.0006*	69.2 (0.8)	66.9 (0.6)	65.9 (0.5)	*<.0001*
**Mean BMI**	27.3 (0.2)	26.2 (0.2)	25.0 (0.2)	*<.0001*	27.8 (0.3)	26.0 (0.2)	25.1 (0.2)	*<.0001*
**Educational level (%)**								
None	11	2	2	*<.0001*	25	10	5	*<.0001*
Primary school completed	26	24	23		31	31	20	
Technical/professional school	20	24	29		9	23	29	
Secondary school	17	13	10		19	18	21	
Longer education (including University degree)	26	37	36		16	18	25	
**Smoking status (%)**								
Never	29	36	39	*0.0003*	72	67	62	*0.01*
Former	41	42	39		11	22	23	
Current	30	22	22		17	11	15	
**Physical activity (%)**								
Inactive	21	16	18	*0.10*	35	28	25	*0.07*
Moderately inactive	33	31	31		37	38	37	
Moderately active	28	25	25		18	20	25	
Active	18	28	26		10	14	13	
**Menopause** (%)								
Pre	-	-	-	-	18	20	23	*0.001*
Peri	-	-	-		69	60	55	
Post	-	-	-		13	20	22	
**Mean total energy intake** (kcal/day)	2550.8 (32.5)	2299.0 (42.4)	2313.5 (49.8)	<.0001	1959.2 (36.4)	1927.8 (27.0)	1936.7 (24.9)	0.90
**Mean total fat** (g/day)	99.8 (1.6)	92.3 (2.1)	94.0 (2.5)	0.009	78.1 (1.9)	76.7 (1.4)	79.8 (1.3)	0.35
**Mean saturated fat** (g/day)	33.0 (0.7)	34.2 (0.9)	36.3 (1.0)	0.006	25.5 (0.8)	27.9 (0.6)	29.6 (0.6)	0.001
**Mean monounsaturated fat** (g/day)	43.3 (0.8)	35.5 (1.1)	33.8 (1.2)	<.0001	33.7 (0.9)	30.1 (0.7)	29.9 (0.6)	0.001
**Mean total polyunsaturated fat** (g/day)	15.3 (0.3)	15.5 (0.4)	17.4 (0.5)	0.005	12.4 (0.4)	12.3 (0.3)	14.2 (0.3)	<.0001
**Mean alcohol intake** (g/day)	25.3 (1.0)	14.7 (1.3)	12.1 (1.5)	<.0001	8.9 (0.7)	7.6 (0.5)	6.6 (0.5)	0.002

^a^p-values from the regression of the considered variable (continuous or categorical) on the log-transformed elaidic acid levels

In men, increasing tertiles of elaidic acid level were associated with a greater likelihood of having a post-graduate education, never smoking, and being active, and with decreasing intakes of energy, total fat (monounsaturated fat) and alcohol. In women, increasing tertiles of elaidic acid level were associated with a greater likelihood of having a post-graduate education, smoking, and with decreasing intake of alcohol and monounsaturated fat.

**Fig 1:**
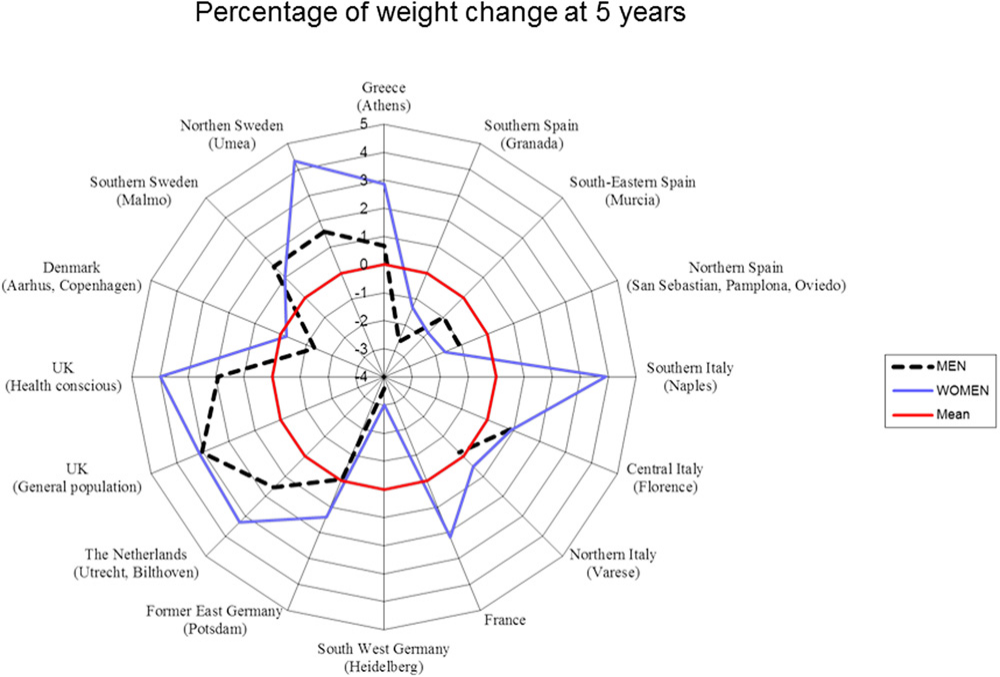
The EPIC regions (n = 16) were ordered from South to North. These geographical regions were designated by grouping some of the 23 EPIC centers together: France (Paris and surroundings), Northern Italy (Varese), Central Italy (Florence), Southern Italy (Naples), Northern Spain (San Sebastian, Navarra, Asturias), South-Eastern Spain (Murcia), Southern Spain (Granada), Greece (Athens and other regions), Northern Sweden (Umeå), Southern Sweden (Malmö), Denmark (Aarhus and Copenhagen), UK (Oxford, the health conscious group, vegans and ovo-lacto-vegetarians), UK (Cambridge, the General population), The Netherlands (Utrecht and Bilthoven), former East Germany (Potsdam), and South-West Germany (Heidelberg).

Fig. 1 shows the center mean weight change (%) at 5 years in men and in women across centers. The median weight change at 5-year follow-up was 0.86% (95% central range = -12.3 to 11.9%) among women and 0.56% (95% central range = -9.2 to 11.1%) among men. The baseline median proportion of elaidic acid (18:1n-9*trans*), the main *trans* fatty acid isomer originating from industrial processing of foods, was 0.18% of total fatty acids among women (95% central range = 0.07–0.39), and 0.12% in men (95% central range range = 0.04–0.34).

**Fig 2:**
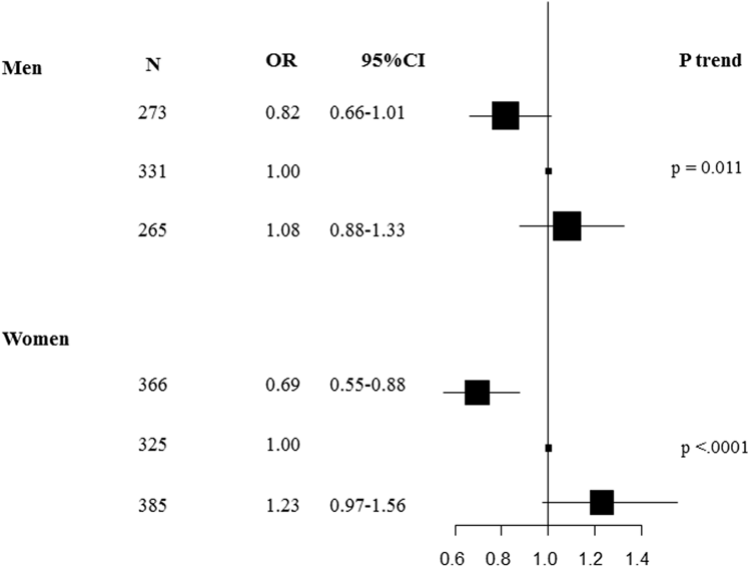
The associations between plasma phospholipid fatty acids and percent of weight change at 5 years were investigated using a multinomial logistic regression model. The percent of weight change at 5 years was estimated as (weightat 5 years minus weight at baseline/weight at baseline)*100, and expressed as a percentage. Weight change during the follow-up was categorized according to tertiles considering the middle category as the reference category (i.e. stable weight at 5 years in %, -1.59;2.83%). The highest tertile (3, weight gain in %, >2.83%) and the lowest (1, weight loss in %, <-1.59%) were compared to the reference category. Exposure variables (fatty acid concentrations 2 log-transformed) were modeled as continuous variables. The model was adjusted by length of follow-up, age, energy and alcohol intakes, smoking status, physical activity, and region. Analyses were carried out for women and men separately.

In women, doubling elaidic acid was associated with a decreased risk of weight loss (odds ratio (OR) = 0.69, 95% confidence interval (CI) = 0.55–0.88, p = 0.002) and a trend was observed with an increased risk of weight gain during the 5-year follow-up (OR = 1.23, 95% CI = 0.97–1.56, p = 0.082) (p-trend<.0001) (Fig. 2). In men, a trend was observed between doubling elaidic acid level and risk of weight loss (OR = 0.82, 95% CI = 0.66–1.01, p = 0.062) while no significant association was found with risk of weight gain during the 5-year follow-up (OR = 1.08, 95% CI = 0.88–1.33, p = 0.454) (Fig. 2).

Besides *trans* elaidic acid, no significant association was found in women or in men between individual levels of saturated fatty acids (palmitic acid, stearic acid, pentadecanoate, heptadecanoate), *cis*-monounsaturated acids (palmitoleic acid, oleic acid), or the sum of elaidic acid and saturated fatty acids and percent of weight change at 5 years (data not tabulated).

## Discussion

This large epidemiological study conducted within the EPIC cohort provides unique data on biomarkers of industrial TFA and their associations with weight change at 5-year follow-up. We showed evidence that doubling elaidic acid level measured at baseline was associated with a decreased risk of weight loss and a trend was observed with an increased risk of weight gain, particularly in women. This association is specific to elaidic acid, as saturated and *cis*-monounsaturated fatty acids were not significantly associated with weight change. In a previous study within the EPIC cohort, no significant association was found between baseline plasma levels of n-3 polyunsaturated fatty acids and subsequent weight change [17].

### Dietary Sources of Elaidic Acid

*Trans*-monounsaturated fatty acids represent a class of exogenous fatty acids, not synthesized by humans, originating either from ruminant animal meat and dairy fat, mostly vaccenic acid and conjugated linoleic acid, or from partially hydrogenated vegetable oils, used as a substitute for saturated fats in some margarines and industrially highly processed foods, mostly elaidic acid [18]. Ultra-processed products, a type of process that has become increasingly predominant, at first in high-income countries, and now in low and middle-income countries, creates ready-to-consume food products that are energy-dense, and contain TFA, sugar and salt [19, 20]. Even if the intake of TFA has decreased in some high-income countries (e.g. Denmark, Norway, Canada), the increasing worldwide consumption of ultra-processed foods implies that certain subgroups of the population in both high, low and middle-income countries can reach high intake of industrial TFA [21].

### *Trans* Fatty Acids and Obesity

We previously showed that elaidic acid concentrations measured in plasma phospholipids were positively correlated to dietary intake of highly processed foods in a cross-sectional study conducted within the EPIC cohort, indicating that plasma phospholipid elaidic acid is a reliable biomarker of highly processed foods [9]. The trend for a positive association that we reported between baseline plasma levels of elaidic acid and weight gain is consistent with data from a cross-sectional study conducted in Costa Rica, showing positive association between adipose tissue levels of 18:2*trans* fatty acids originating mostly from partially hydrogenated oils, and BMI, while a negative association was found between total 18:1*trans* and BMI, suggesting that specific TFA isomers may have divergent effects on adiposity [22]. However, a recent cohort study conducted in Denmark found no significant association between baseline adipose tissue levels of total TFA or TFA from dairy foods and weight change years after enrolment [23]. Some studies support that this association between industrial TFA and adiposity may be causal [24, 25]. Industrial TFA isomers serve as ligands for the peroxisome proliferator-activated receptors-γ system and can exert a biological effect that promotes abdominal obesity [24]. A randomized trial in monkeys showed evidence that supplementation with industrial TFA isomers induce weight gain and abdominal adiposity, with evidence that there is impaired insulin receptor binding signal transduction [25]. Growing evidence for this specific effect is particularly concerning given the worldwide obesity pandemic and high contents of industrially produced TFA in many foods toward children.

This study has some limits. Firstly, biomarkers of fatty acids are available only at baseline, and the possibility that changes in plasma phospholipid fatty acid levels might have changed during the follow-up is not known. Secondly, BMI, especially when the measure is based on self-reported height and weight, is an insensitive measure of body fat compared with more sensitive and direct approaches such asDEXA. Thirdly, this study was limited by the inability to resolve TFA isomers other than elaidic acid. Finally, as in other observational studies, we cannot rule out the possibility that the association we observed resulted from confounding bias, although we adjusted for known factors related to elaidic acid.

In conclusion, these data suggest that a high intake of TFA originating from industrial processing of foods is associated with decreased risk of weight loss, particularly in women. Further research is needed to explore the causality of the associations and the underlying biological plausibility. Prevention of obesity should consider limiting the consumption of highly processed foods, the source of industrially-produced TFA. Particularly, existing industrial processes generating TFA (partially hydrogenated vegetable oils) should be curbed, as undertaken in some countries—Denmark, Canada, and New York City for a few years now. This evidence is highly relevant and provides a strong rationale for obesity prevention in populations that have experienced the nutritional transition.

## References

[1] Malik VS, Willett WC, Hu FB (2013) Global obesity: trends, risk factors and policy implications Nat Rev Endocrinol 9:13–27. 10.1038/nrendo.2012.199 23165161

[2] Popkin BM, Adair LS, Ng WS (2012) Now and then: The global nutrition transition: The pandemic of obesity in developing countries. Nutr Rev 70:3–21. 10.1111/j.1753-4887.2011.00456.x 22221213 PMC3257829

[3] Astrup A, Dyerberg J, Selleck M, Stender S (2008) Nutrition transition and its relationship to the development of obesity and related chronic diseases. Obes Rev 9:48–52. 10.1111/j.1467-789X.2007.00438.x 18307699

[4] De Ferranti S, Mozaffarian D (2008) The perfect storm: obesity, adipocyte dysfunction, and metabolic consequences. Clin Chem 54:945–55. 10.1373/clinchem.2007.100156 18436717

[5] Melanson E, Astrup A, Donahoo WT (2009) The relationship between dietary fat and fatty acid intake and body weight, diabetes, and the metabolic syndrome. Ann Nutr Metab 55:229–43. 10.1159/000229004 19752544

[6] Thompson AK, Minihane AM, Williams CM (2011) Trans fatty acids and weight gain. Int J Obes 35:315–24. 10.1038/ijo.2010.141 20644558

[7] Saadatian-Elahi M, Slimani N, Chajès V, Jenab M, Goudable J, et al (2009) Plasma phospholipid fatty acid profiles and their association with food intakes: results from a cross-sectional study within the European Prospective Investigation into Cancer and Nutrition. Am J Clin Nutr 89:331–46. 10.3945/ajcn.2008.26834 19056549

[8] Baylin A, Campos H (2006) The use of fatty acid biomarkers to reflect dietary intake. Curr Opin Lipidol 17:22–27. 16407712 10.1097/01.mol.0000199814.46720.83

[9] Chajès V, Biessy C, Byrnes G, Deharveng G, Saadatian-Elahi M, et al (2011) Ecological-level associations between highly processed food intakes and plasma phospholipid elaidic acid concentrations: results from a cross-sectional study within the European Prospective Investigation into Cancer and Nutrition (EPIC). Nutr Cancer 63:1235–50. 10.1080/01635581.2011.617530 22043987

[10] Bingham S, Riboli E (2004) Diet and cancer—the European Prospective Investigation into Cancer and Nutrition. Nat Rev Cancer 4:206–15. 14993902 10.1038/nrc1298

[11] Riboli E, Kaaks R (1997) The EPIC Project: rationale and study design. European Prospective Investigation into Cancer and Nutrition. Int J Epidemiol 26 Suppl 1:S6–14. 9126529 10.1093/ije/26.suppl_1.s6

[12] Besson H, Ekelund U, Luan J, May AM, Sharp S, et al (2009) A cross-sectional analysis of physical activity and obesity indicators in European participants of the EPIC-PANACEA study. Int J Obes (London) 33:497–506. 10.1038/ijo.2009.25 19223851

[13] Slimani N, Kaaks R, Ferrari P, Casagrande C, Clavel-Chapelon F, et al (2002) European Prospective Investigation into Cancer and Nutrition (EPIC) calibration study: rationale, design and population characteristics. Public Health Nutr 5(6B):1125–45. 12639223 10.1079/PHN2002395

[14] Slimani N, Deharveng G, Charrondière RU, van Kappel AL, Ocké MC, et al (1999) Structure of the standardized computerized 24-h diet recall interview used as reference method in the 22 centers participating in the EPIC project. European Prospective Investigation into Cancer and Nutrition. Comput Methods Programs Biomed 58:251–66. 10094230 10.1016/s0169-2607(98)00088-1

[15] Haftenberger M, Lahmann PH, Panico S, Gonzalez CA, Seidell JC, et al (2002) Overweight, obesity and fat distribution in 50- to 64-year-old participants in the European Prospective Investigation into Cancer and Nutrition (EPIC). Public Health Nutr 5:1147–62. 12639224 10.1079/PHN2002396

[16] Chajès V, Hulten K, van Kappel AL, Winkvist A, Kaaks R, et al (1999) Fatty acid composition in serum phospholipids and risk of breast cancer: an incident case-control study in Sweden. Int J Cancer 83: 585–90. 10521790 10.1002/(sici)1097-0215(19991126)83:5<585::aid-ijc2>3.0.co;2-z

[17] Jakobsen MU, Dethlefsen C, Due KM, Slimani N, Chajès V, et al (2011) Plasma phospholipid long-chain n-3 polyunsaturated fatty acids and body weight change. Obes Facts 4:312–18. 10.1159/000330710 21921655 PMC6444826

[18] Sommerfeld M (1983) Trans unsaturated fatty acids in natural products and processed foods. Prog Lipid Res 22:221–33. 6356151 10.1016/0163-7827(83)90010-3

[19] Slimani N, Deharveng G, Southgate DA, Biessy C, Chajès V, et al (2009) Contribution of highly processed foods to the nutrient intakes and patterns of middle-aged populations in the European Prospective Investigation into Cancer and Nutrition study. Eur J Clin Nutr 63:S206–25. 10.1038/ejcn.2009.82 19888275

[20] Monteiro CA, Moubarac JC, Cannon G, Ng SW, Popkin B (2013) Ultra-processed products are becoming dominant in the global food system. Obes Rev 14:21–8. 10.1111/obr.12107 24102801

[21] Stender S, Dyerberg J, Astrup A (2006) High levels of industrially produced trans fat in popular fast foods. N Engl J Med 354:1650–52. 16611965 10.1056/NEJMc052959

[22] Smit LA, Willett WC, Campos H (2010) Trans fatty acid isomers in adipose tissue have divergent associations with adiposity in humans. Lipids 45:693–700. 10.1007/s11745-010-3442-z 20628829 PMC2922622

[23] Hansen CP, Berentzen TL, Østergaard JN, Dahm CC, Hellgren LI, et al (2014) Adipose tissue trans-fatty acids and changes in body weight and waist circumference. Br J Nutr 111:1283–91. 10.1017/S0007114513003747 24286469

[24] Mozaffarian D, Katan MB, Ascherio A, Stampfer MJ, Willett WC (2006) Trans fatty acids and cardiovascular disease. N Eng J Med 354:1601–13. 16611951 10.1056/NEJMra054035

[25] Kavanagh K, Jones KL, Sawyer J, Kelley K, Carr JJ, et al (2007) Trans fat diet induces abdominal obesity and changes in insulin sensitivity in monkeys. Obesity 15:1675–84 17636085 10.1038/oby.2007.200

